# EFFECTS AND DIRECTIONALITY OF A HANDICRAFT INTERVENTION ON HAND FUNCTION AND HIGH-LEVEL COGNITION IN OLDER ADULTS

**DOI:** 10.1093/geroni/igae098.0263

**Published:** 2024-12-31

**Authors:** Kimi Kobayashi-Cuya, Susumu Ogawa, Ai Iizuka, Tomoya Takahashi, Hiroyuki Suzuki

**Affiliations:** Tokyo Metropolitan Institute for Geriatrics and Gerontology, Tokyo, Japan; Tokyo Metropolitan Institute for Geriatrics and Gerontology, Tokyo, Japan; Tokyo Metropolitan Institute for Geriatrics and Gerontology, Tokyo, Japan; Tokyo Metropolitan Institute for Geriatrics and Gerontology, Tokyo, Japan; Tokyo Metropolitan Institute for Geriatrics and Gerontology, Tokyo, Japan

## Abstract

A longitudinal bidirectional association of hand dexterity with executive function and processing speed in high-functioning older adults has been reported, suggesting that community-level intervention programs using activities that demand dexterous abilities may help prevent the decline in these cognitive functions. Therefore, the present single-blind crossover randomized controlled trial examined the intervention effects of a 12-week manual dexterity program in community-dwelling older adults on hand sensorimotor and high-level cognitive functions, and assessed the direction of their associations. Fifty-three participants (mean age±SD, 75.2±5.6, 83% female) were randomly assigned to the intervention (handicrafts and creative cooking) or active control period (lectures on health maintenance). The primary outcome was hand dexterity, and the secondary outcomes were handgrip strength and hand sensation for hand function, and for high-level cognition, executive function, which was assessed with the trail making test (TMT)B, ∆TMT (TMTA-B) and the letter and category fluency tests (LFT and CFT, respectively), and processing speed, evaluated with TMTA and digit symbol. Linear mixed-effects models, testing for carry-over effects, showed intervention effects in hand dexterity (p< 0.05) and executive function (TMTB, ∆TMT and LFT, p< 0.05), whereas no effects were found for handgrip, hand sensation, nor processing speed. Also, cross-lagged panel models, adjusting for potential confounders, further revealed moderate and significant associations between baseline hand dexterity and subsequent increase in TMTB, LFT (p< 0.001) and ∆TMT (p< 0.05). Our results indicate that the implementation of a short-term manual dexterity program may contribute to the prevention of decline in executive function in community-dwelling older adults.

